# Reversible naftifine-induced carotenoid depigmentation in *Rhodotorula mucilaginosa* (A. Jörg.) F.C. Harrison causing onychomycosis

**DOI:** 10.1038/s41598-017-11600-7

**Published:** 2017-09-11

**Authors:** Augustin C. Moț, Marcel Pârvu, Alina E. Pârvu, Oana Roşca-Casian, Nicoleta E. Dina, Nicolae Leopold, Radu Silaghi-Dumitrescu, Cristina Mircea

**Affiliations:** 1 Babeș-Bolyai University, Faculty of Chemistry and Chemical Engineering, 11 Arany Janos Street, 400028 Cluj-Napoca, Romania; 2 Babeș-Bolyai University, Faculty of Biology and Geology, 42 Republicii Street, 400015 Cluj-Napoca, Romania; 3 Iuliu Hatieganu University of Medicine and Pharmacy, Faculty of Medicine, Department of Pathophysiology, 3 Victor Babes Street, 400012 Cluj-Napoca, Romania; 4 Babeș-Bolyai University, Alexandru-Borza Botanical Garden, 42 Republicii Street, 400015 Cluj-Napoca, Romania; 5 National Institute for Research and Development of Isotopic and Molecular Technologies, 67–103 Donath Street, 400293 Cluj-Napoca, Romania; 6 Babeș-Bolyai University, Faculty of Physics, 1 Mihail Kogalniceanu Street, 400084 Cluj-Napoca, Romania

## Abstract

*Rhodotorula mucilaginosa* was isolated from a patient with onychomycosis, and identification was confirmed by morphological and cultural characteristics as well as by DNA molecular analysis. Antifungal agents naftifine (10 mg/mL, active substance in Exoderil) and bifonazole (10 mg/mL, active substance in Canespor) were tested in different concentrations to assess *in vitro* effects on fungal growth and carotenoid synthesis. The antifungal mechanisms of action of naftifine and bifonazole against *R. mucilaginosa* isolates were similar and affected the biosynthetic pathway of ergosterol. For the first time, this research demonstrates that naftifine affects the carotenoid biosynthetic pathway, producing depigmentation of *R. mucilaginosa* in solid and liquid media. Furthermore, depigmentation was a reversible process; naftifine-treated yeast cells that were depigmented resumed carotenoid production upon transfer to fresh media. Raman and UV-vis spectrophotometry in conjunction with chromatographic analysis detected changes in carotenoids in yeast cells, with torulene decreasing and B-carotene increasing after repigmentation. Transmission electron micrographs revealed critical ultrastructural modifications in the depigmented cells after naftifine treatment, i.e., a low-electron-density cell wall without visible mucilage or lamellate structure.

## Introduction

Onychomycosis is a multifactorial nail fungal infection^1–3^. It is an important public health problem because of its high prevalence, high rates of recurrence and progression to chronic lesions^4^. Onychomycosis accounts for 50% of all nail diseases. It affects 2–13% of the general population, and this percentage increases with age, reaching up to 40% in the elderly^5^. Thickened, discoloured, deformed nails without pain are the most common symptoms of a fungal nail infection. Although the clinical picture can be very suggestive, the diagnosis should be confirmed by a routine method before starting treatment^5^. The most commonly used methods are direct potassium hydroxide (KOH) examination, culture, and, to a lesser extent, nail biopsy. The diagnosis of onychomycosis is made when one or more of the three diagnostic tests are positive. Other tests are expensive and require the use of specialized equipment and materials^6^.

Common pathogens in onychomycosis are dermatophytes, nondermatophyte moulds (NDMs) and yeasts^1, 3, 7^. The nomenclature of fungal infections proposed by the International Society for Human and Animal Mycology suggests that the term onychomycosis should be replaced by tinea unguium when the aetiological agent is a dermatophyte; by onyxis when yeasts are the cause; by ungual candidiasis when the agent is *Candida*; and by ungual mycosis when the causal agent is an NDM^8^.

Most cases of tinea unguium are caused by dermatophytes that belong to three genera: *Trichophyton*, *Microsporum*, and *Epidermophyton*. The fungi *Trichophyton rubrum*, *T. mentagrophytes*, and *Epidermophyton floccosum* are the most common aetiologic agents of onychomycosis worldwide. The most commonly described species of NDM are *Scopulariopsis brevicaulis*, *Fusarium* spp., *Acremonium* spp., *Aspergillus* spp., *Scytalidium* spp., and *Onychocola canadienses* ^8^. *Candida* species are the most frequent among the yeasts^7^. Other yeasts accepted in recent years as causative agents of onychomycosis are *Rhodotorula mucilaginosa* ^9^, *R. minuta* ^10^ and *R. glutinis* ^11^. Several authors have isolated *Rhodotorula* from ecosystems and environments^12–15^, humans^16–18^ and animals^13^. During the last few decades, they have emerged as opportunistic pathogens, particularly in immunocompromised patients^19^.

Because onychomycosis aetiologic agents have variable susceptibility to antifungal drugs, laboratory diagnosis is a necessary tool for ascertaining the best therapeutic option^4^. Precise and correct identification of the species responsible for onychomycosis in a given patient is required to select the most appropriate treatment as well as to avoid the misuse of antifungals^9^.

Thus, *in vitro* susceptibility tests demonstrated that *R. mucilaginosa* is sensitive to low concentrations of amphotericin B and 5-flucytosine and resistant to high concentrations of fluconazole, itraconazole, voriconazole and terbinafine^9^. Additionally, in the case of *R. glutinis*, minimum inhibitory concentrations (MIC) vary depending on the antifungal drug: 0.125 *μ*g/mL for itraconazole; 0.5 *μ*g/mL for amphotericin B, anidulafungin and posaconazole; 1 *μ*g/mL for voriconazole; 16 *μ*g/mL for caspofungin and 128 *μ*g/mL for fluconazole^11^. Overall, for humans the use of antifungals against *Rhodotorula* infections is still controversial because *in vitro* some are inactive against the majority of clinical isolates of *Rhodotorula* spp.^19^.

*R. mucilaginosa* is a basidiomycetous yeast. It presents only spheroidal to oval budding cells (2.5–6.5 × 6.5–14.0 *µ*m) with carotenoid pigments^12, 20^ and without the rudimentary formation of hyphae^21, 22^. This species is characterized by yeast-like colonies that are coral pink and moist to mucoid on Sabouraud Dextrose Agar (SDA), by the inability to assimilate inositol and by the absence of fermentation^23^. Carotenoids can act as vitamin A precursors, having colouring and antioxidant properties^24^. Carotenoids are widely produced pigments found in algae, yeasts and plants. Their main functions are reactive oxygen species scavenging and protection against photooxidative damage. Four main pigments, torularhodin, torulene, beta-carotene and gamma-carotene, are synthesized by *R. mucilaginosa* species^24, 25^, and carotenoid production is usually enhanced by stress factors such as UV exposure, oxidative stress or osmotic stress^26^. Torularhodin was shown to be correlated with the survival of the cells under UV-B light^27^. Beta-carotene and torularhodin also showed antioxidant activity, preventing hyperoxia-induced cytotoxicity^28^.

Carotenoid synthesis starts from acetyl-CoA that is transformed into isopentenyl pyrophosphate and further to lycopene, which is the source of many other carotenoids^29^. Cases of depigmentation have previously been reported and are thought to be induced by chemicals interfering with the biosynthetic process. Naftifine, a topical allylamine, has been shown to interfere with the pigmentation process of *Staphylococcus aureus* colonies by specifically and strongly inhibiting isoprenoid synthesis^30^.

The study aimed first to find onychomycosis etiology and sensitivity to naftifine and bifonazole. Secondly, *R. mucilaginosa* growth, carotenoids metabolism and structural changes were analysed.

## Results and Discussion

**Figure 1:**
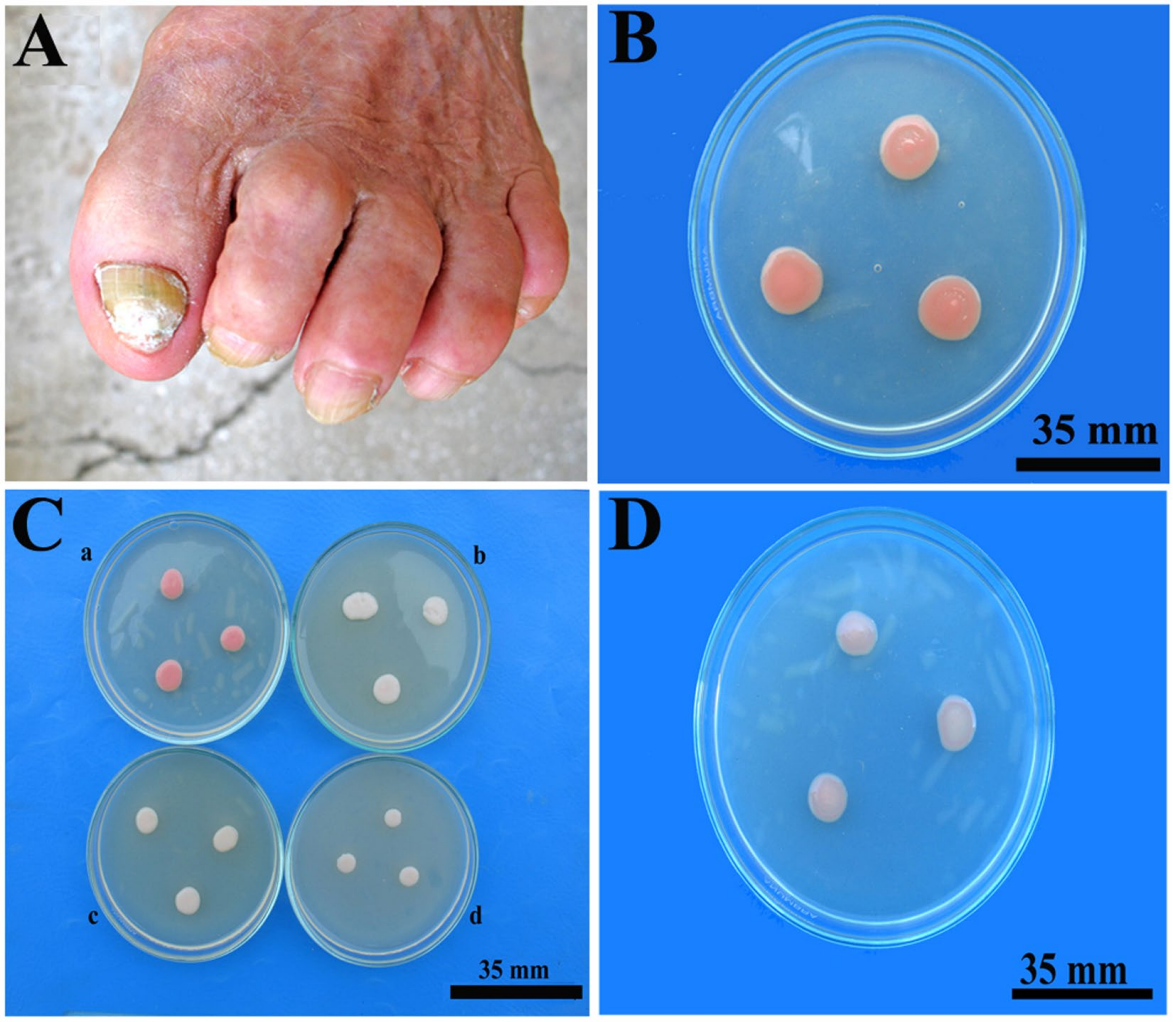
(**A**) Toenails affected by onychomycosis; (**B**). *Rhodotorula mucilaginosa* control colonies on Sabouraud Dextrose Agar (SDA); (**C**). *R. mucilaginosa* control colonies (a) and depigmented colonies obtained by including the fungal inoculum in 1 mg/mL naftifine solution for 10 minutes before inoculation (b), depigmented colonies obtained on SDA with 0.1 mg/mL naftifine (c) and 0.2 mg/mL naftifine (d); (**D)**. Transfer and growth of depigmented yeast cells to fresh SDA results in repigmentation.

The results indicated that *R. mucilaginosa* was a causative agent in this specific case of onychomycosis in an aged patient with chronic HBV hepatitis (Fig. 1A). After incubation of all toenail samples in SDA control media, *R. mucilaginosa* isolates were identified by morphological and cultural characteristics. Control colonies of *R. mucilaginosa* obtained on SDA were mucoid, with red colour (Fig. 1B) and a diameter of 12-13 mm 3 days after inoculation. Molecular analysis was performed for species identity confirmation^14, 23^. The DNA sequence was run against the BLAST-NCBI nucleotide database^31^ as a query and matched identically with *R. mucilaginosa* (GenBank: KU052792.1) species.

Then, by exposing a control *R. mucilaginosa* isolate for 10 minutes to 1 mg/mL naftifine solution, depigmented fungal colonies were obtained on SDA control media. The depigmented colonies were mucoid and had similar diameters to the red colonies but showed a milky colour. Moreover, by including small concentrations of naftifine (0.1 mg/mL, 0.2 mg/mL) in SDA before inoculation of *R. mucilaginosa* isolate, an inhibitory effect was obtained compared to the control colonies (Fig. 1C). These depigmented colonies had a diameter of 7-8 mm on SDA with 0.1 mg/mL naftifine and of 5-6 mm on SDA with 0.2 mg/mL naftifine, showing a small dose-dependent inhibitory effect of the drug (Fig. 1C). Furthermore, by inoculating depigmented *R. mucilaginosa* isolate into SDA control media, colonies regained pigment (Fig. 1D). Considering that carotenoids are important substances against reactive oxygen species (ROS), depigmentation may be correlated with increased oxidative stress and fungal injury. Repigmentation suggested that the carotenoid metabolism changes induced in the fungi by naftifine were reversible. These surprising results suggested further analysis regarding *R. mucilaginosa* carotenoid metabolism and related structural changes.

Preliminary tests of *R. mucilaginosa* growth under our conditions indicated that the stationary phase was reached after 60–70 h. However, in the initial exponential stage the cells appeared mostly colourless (low carotenoid content), and only after approximately 50 h could the pigmentation be observed. This monitoring was performed at various pH values (pH range 3.5–7.5), especially for tracking carotenoid formation. The growth was pH-dependent only in the initial exponential phase (Fig. S1A). Although the pH profile change in time was dependent on the initial pH (Fig. S1B), indicating a pH-dependent metabolism for *R. mucilaginosa*, carotenoid formation in terms of both rate and composition did not depend on pH significantly, as observed by monitoring the intensity and spectral features from the resonant Raman spectra (Fig. S2). We aimed to further understand the factors that influenced and controlled carotenoid formation in *Rhodotorula* spp. beyond the factors that interfered with basic metabolism, such as type of C and N sources and their ratio^26^, compounds inducing ROS stress (e.g., duroquinone, methylene blue). ROS upregulated carotenoid content in *R. mucilaginosa*, indicating that the carotenoids were a response of the cells to the negative effects of ROS agents (e.g., superoxide, singlet oxygen) as previously shown^28, 32^. Other chemicals, such as amphotericin and diphenylamine, not only inhibited carotenoid formation but also cell development (Fig. S3). In addition, we hypothesized that the photosensitizers hypericin and hyperforin could also induce carotenoid formation; however, this was not the case (Fig. S3), most likely due to the low level of the induced potential stress.

**Figure 2:**
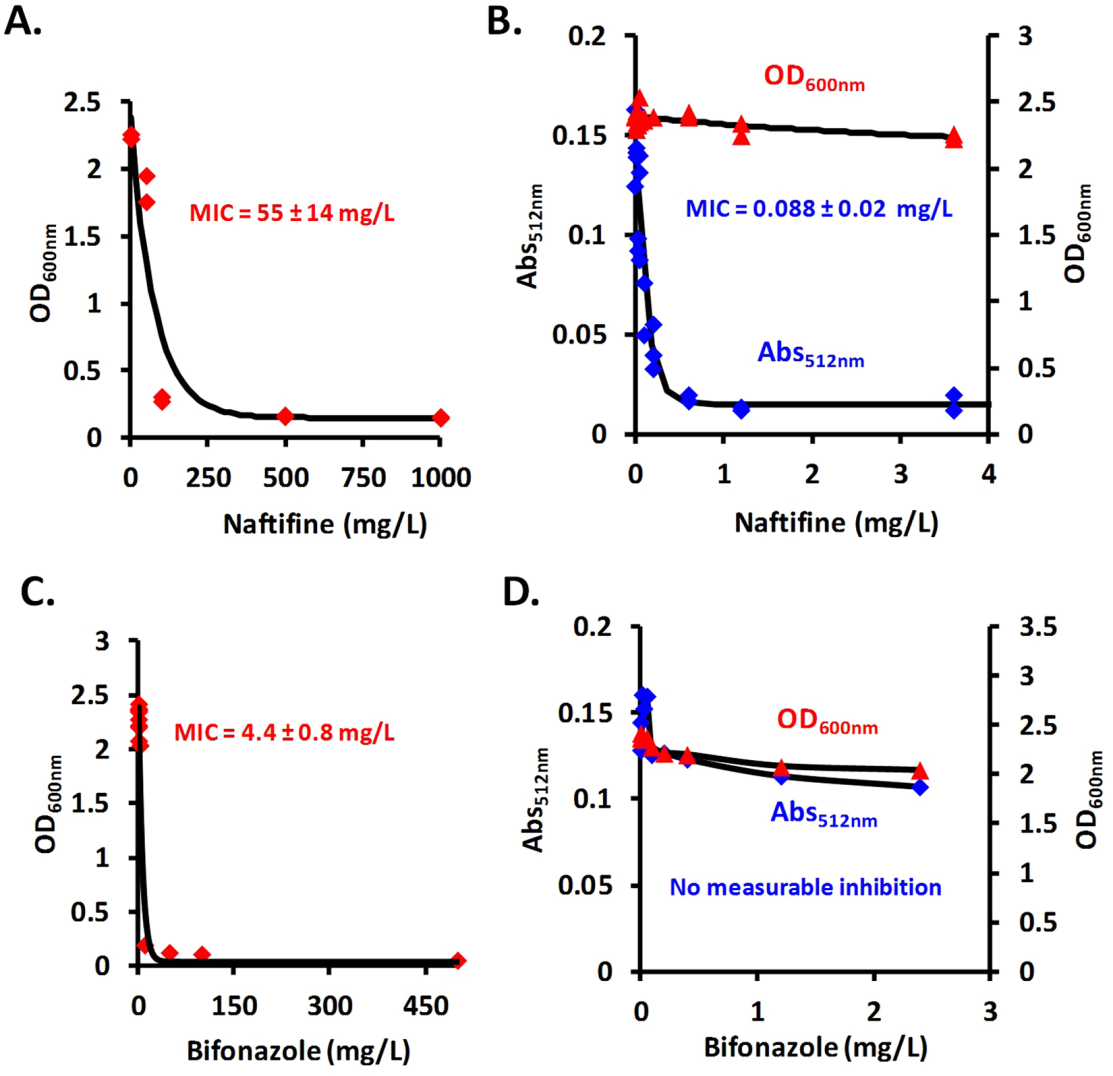
(**A**) Determination of MIC50 values for naftifine content in the pharmaceutical product Exoderil by monitoring the OD at 600 nm and (**B)** by absorbance at 512 nm after cell disruption with DMSO. (**C)** Determination of MIC50 values for bifonazole content in the pharmaceutical product Canespor by monitoring the OD at 600 nm and (**D**) by absorbance at 512 nm after cell disruption with DMSO.

During these initial experiments, we observed that one of the two tested antifungal agents, namely, naftifine (an allylamine antifungal drug), did not inhibit cell growth at lower concentrations, but rather induced depigmentation of the cells when growing on solid medium (Fig. 1C). The same results were obtained in liquid media, in controlled conditions. In addition to determination of naftifine MIC50 (55 ± 14 mg/L) for its antifungal activity (Fig. 2A), quantified by OD_600nm,_ the effect of naftifine on depigmentation was assessed by OD_512nm_ and a corresponding MIC50 (0.088 ± 0.02 mg/L) was determined (Fig. 2B). Depigmentation did not affect the cell growth at these naftifine concentrations, as could be observed by the optical density values. In contrast, bifonazole (an imidazole antifungal drug) did not produce any depigmentation in *R. mucilaginosa*, although its MIC50 for antifungal activity was one order of magnitude lower, 4.4 ± 0.8 mg/L (Fig. 2C and D).

**Figure 3:**
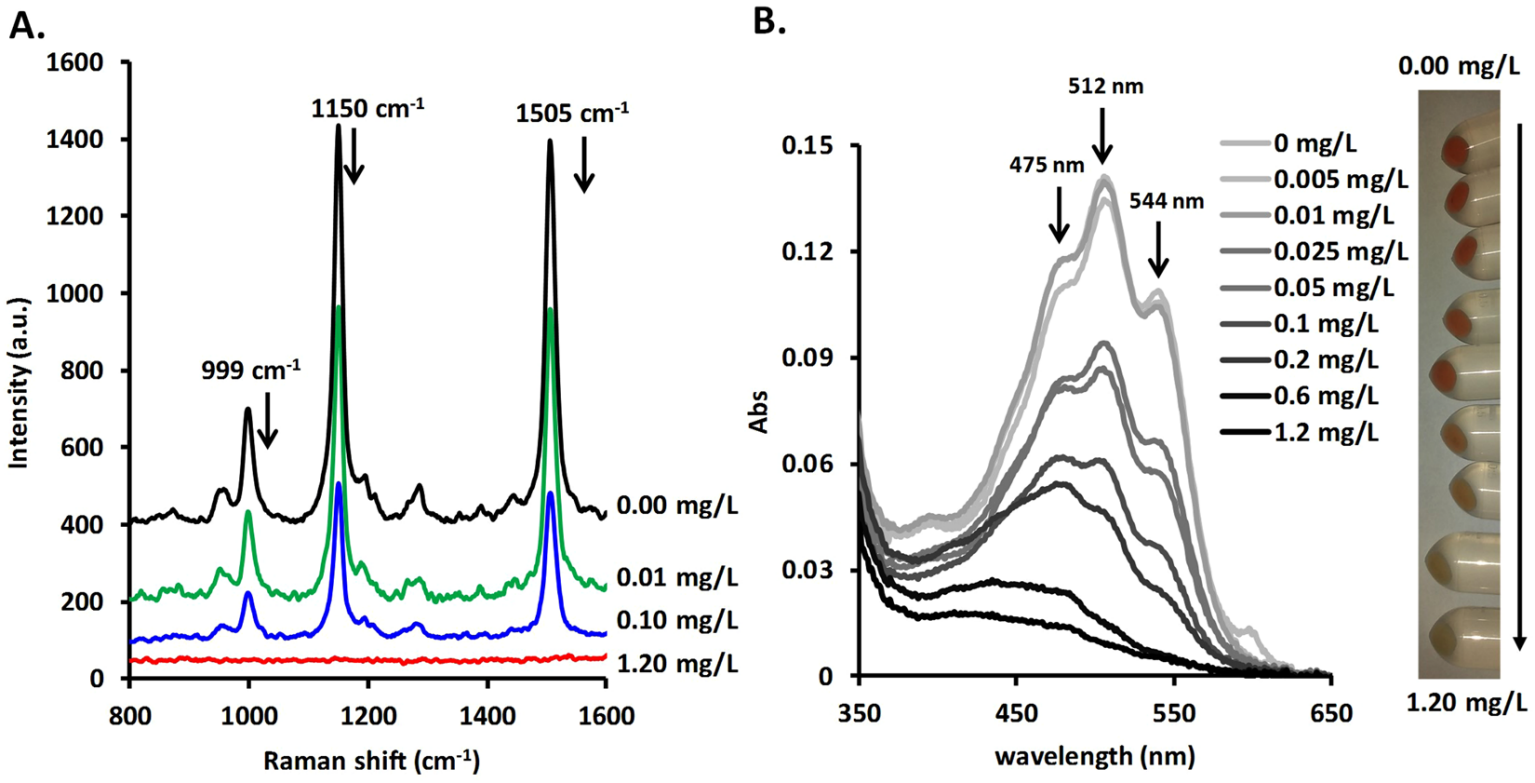
(**A)** Resonant Raman spectra of *R. mucilaginosa* cells grown in media containing various naftifine concentrations (indicated to the right of each spectrum). (**B**) Concentrationdependent UV-vis spectra of DMSO-disrupted *R. mucilaginosa* cells after growth in naftifinecontaining media. On the right, an image of the cell pellets at increasing naftifine concentrations after washing and before disruption is shown.

Similar depigmentation was observed by resonant Raman spectroscopy (Fig. 3A), where besides the expected decrease in signal intensities of the main peaks 999 cm^−1^ (C = CH deformation), 1150 cm^−1^ (symmetrical C-C stretching), 1505 cm^−1^ (symmetrical C = C stretching) due to carotenoid content, subtle changes could be observed in the 1200–1400 cm^−1^ region. Minor Raman shifts were recorded for the secondary peak at 1283 cm^−1^ (torulene), ascribed to the CH_2_ deformation in carotenoids^33, 34^. This band was shifted in the case of torularhodin at 1284 cm^−1^, at 1286 cm^−1^ for β-carotene and at 1290 cm^−1^ for unidentified carotenoid (C_nd)_, indicating a possible change of the carotenoid composition in the cells, thus a qualitative change. This observation was further supported by the UV-vis spectra, where in addition to an absorbance decrease with increased naftifine concentrations, the spectral profiles also changed (i.e., the ratio of the three main spectral features) (Fig. 3B).

**Figure 4:**
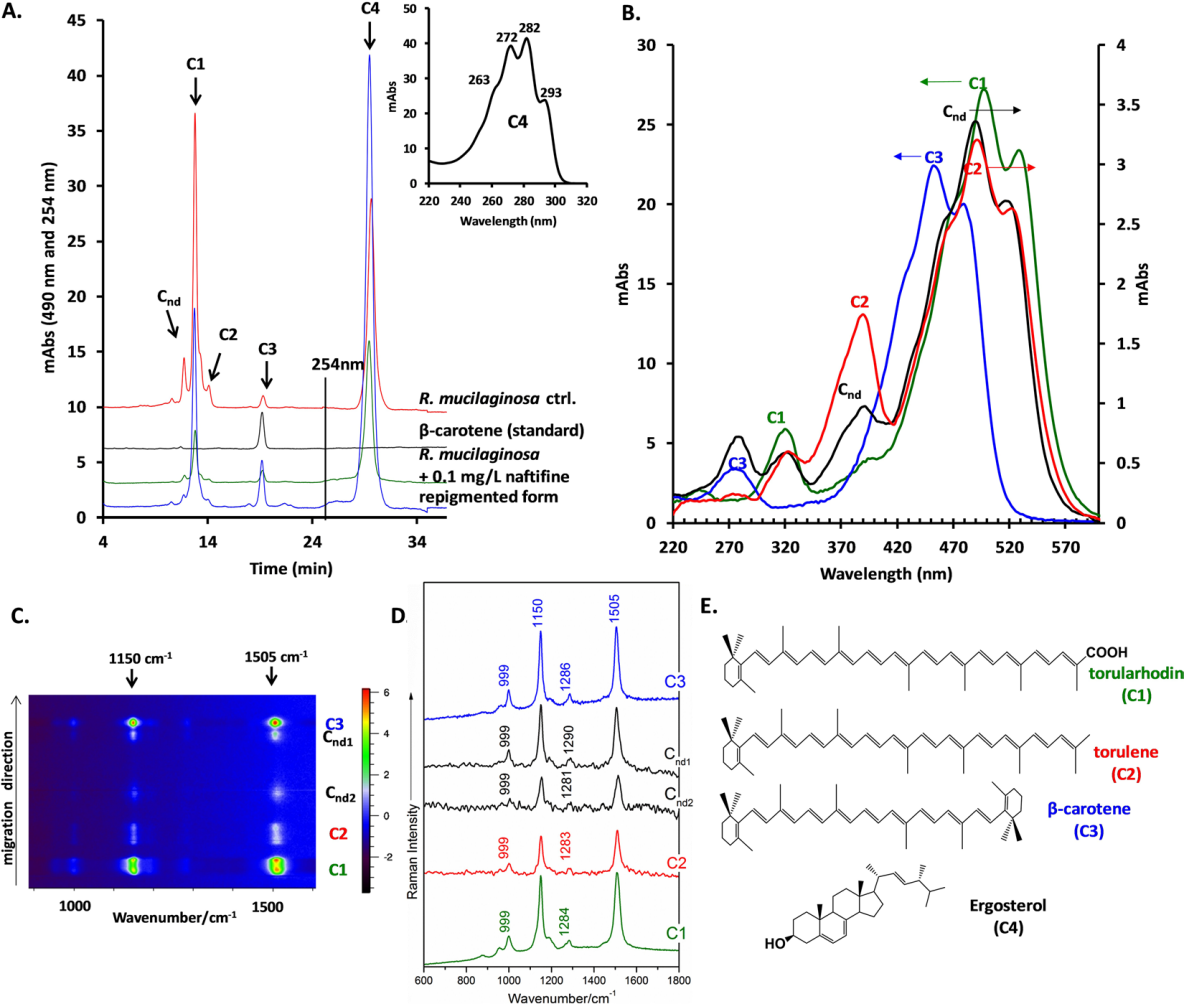
(**A**) HPLC-DAD chromatograms of the DMSO extracts of *R. mucilaginosa* (control, grown in the presence of 0.1 mg/L naftifine and repigmented) as well as β-carotene standards. The major peaks are indicated by C1-C4 labels and Cnd (unidentified compound). The chromatogram was monitored by mAbs at 490 nm for 25 minutes (carotenoid detection) and 254 nm from that point on. In the inset, the spectrum of C4 (ergosterol) is shown. (**B**) UV-vis spectra of the identified compounds. The arrows indicate the corresponding y-axis. (**C**) Raman signal contour map obtained by mapping the HPTLC chromatogram containing the carotenoid-specific components (indicated on the right along with the intensity colour code scale). (**D**) Individual Raman signals of the HPTLC separated components. (**E)** Chemical structures of the identified compounds.

Next, for more in-depth investigations, chromatographic analysis of the extracted carotenoids was performed. From a variety of solvents or conditions for cell disruption available in the literature, we chose simple DMSO treatment of the washed cells. The advantages of this procedure are the high yield of extraction (only a colourless pellet remained, indicating a quantitative extraction) and the good solubility of ergosterol in this solvent, which allowed a simultaneous extraction of both type of constituents. The High-Performance Liquid Chromatography (HPLC) analysis allowed the separation of four main carotenoids including torularhodin, torulene and β-carotene (C1, C2 and C3, respectively), in good agreement with previous data^27, 32^ (Fig. 4A), while the other compound (C_nd_) could not be identified. The identification was done using a pure standard for β-carotene and retention times, as well as using highly specific spectral features of the other two compounds, which were in very good agreement with already known data (Fig. 4B, Table 1). The unidentified compound presented torulene-like carotenoid absorption spectral features and had a chromatographic behaviour (lower retention time allowing assignment as hydroxy-torulene) resembling previously detected carotenoids in other *Rhodotorula* spp.^35^. Additionally, ergosterol was also separated using the same method and identified based on its chromatographic and highly specific spectral characteristics (Fig. 4A). Moreover, using a dedicated method for ergosterol extraction, a highly similar spectrum was obtained. Therefore, all these related components could be quantitatively evaluated, allowing a comparative analysis for cells growing in different conditions. Thin-Layer Chromatography (TLC) was also used for carotenoid separation after DMSO removal using extraction cartridges. Using this method, the same three components were separated and identified, followed by their Raman detection directly on the plate. The contour map of the detected signal is presented in Fig. 4C, and the individual Raman signals are shown in Fig. 4D. As expected, the spectra had a high degree of similarity due to highly similar chemical structures (Fig. 4E); nevertheless, subtle changes could be observed at approximately 1280 cm^−1^, and a slight spectral shift appeared at 1505 cm^−1^ band (Table 1, Fig. 4D). In addition to torularhodin, torulene and β-carotene, two unidentified carotenoids were also observed (probably different than the one from HPLC analysis, due to retention time analysis). This may have occurred because in the introduction of an extra sample preparation step (cartridge extraction for DMSO removal), analyte alteration could occur, especially for those present in lower quantities.

**Table 1:** Chromatographic and spectral characteristics of the separated compounds.

Compound	UV-vis absorption maxima as determined (nm)^a^			UV-vis absorption maxima of standards^35, 50^ (nm)			Rt (min)	Rf	Resonant Raman maxima as determined (cm^−1^)		
C_nd_ (torulene-like)	461	488	517	461	487	518	11,71	nd	nd	nd	nd
C1 (torularhodin)	471	498	526	473	498	525	12,79	0,46	1511	1284	1149
C2 (torulene)	461	489	518	460	485	516	14,14	0,52	1508	1283	1151
C3 (β-carotene)	428	454	477	430	452	478	18,8	0,92	1505	1286	1150
C4 (ergosterol)	272	282	293	272	282	293	29,25	nd	nd	nd	nd

^a^The solvent strongly influences the spectrum profile; therefore, for reference, a solvent with similar polarity had to be used for a proper comparison. nd = not detected.

**Figure 5:**
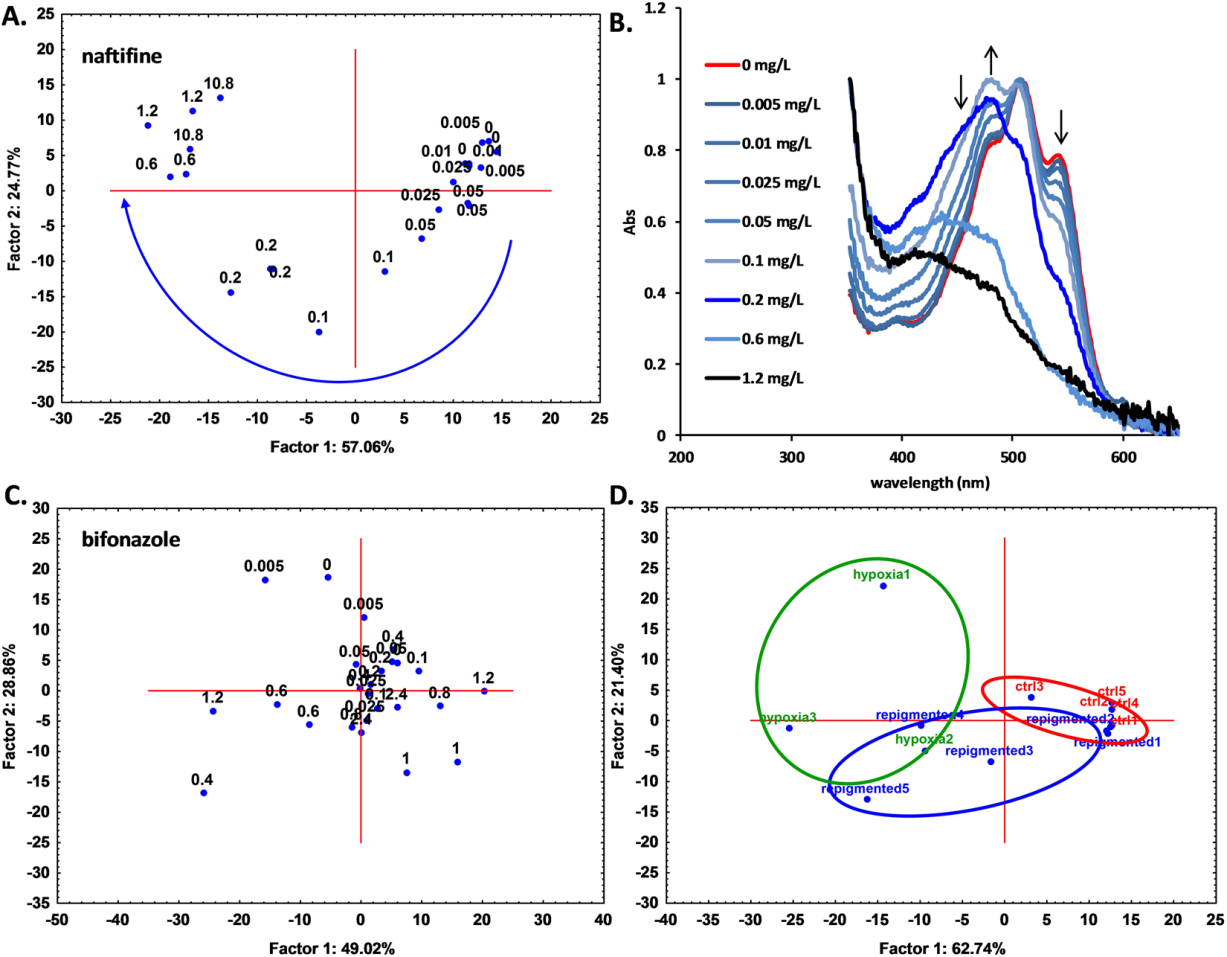
(**A)** Scatterplot of the first two principal component scores obtained after applying PCA to the UV-vis spectra of DMSO-disrupted *R. mucilaginosa* cell extract. Cells were grown at nine levels of naftifine concentrations (0 to 1.2 mg/L, indicated on the plot) in triplicate. The curved arrow indicates the position shift in the graph as the naftifine concentration increases. (**B)** The averaged UV-vis spectra used in the abovementioned PCA analysis at specific working concentrations. The arrows indicate spectral shifts as the naftifine concentration increases. (**C)** Scatterplot obtained identically to A, but with bifonazole used instead of naftifine. (**D**) Scatterplot of the first two principal component scores obtained after applying PCA to the UV-vis spectra of DMSO-disrupted *R. mucilaginosa* cell extract. Cells were grown in standard conditions (control, ctrl.), after repigmentation (in quintuplicate) and in hypoxic conditions (in triplicate).

A Principal Component Analysis (PCA) was applied to the absorbance spectra of the DMSO extracts of the cells grown under various naftifine concentrations, and the plot of the scores corresponding to the first two principal components are shown in Fig. 5A. Surprisingly, two different clusters were noted, one with samples at low concentrations and one with samples at high concentrations, together with a transition zone. Such a grouping indicated different compositions of carotenoids with increasing naftifine concentration. To eliminate the concentration effect (decrease in intensity as the carotenoid content is diminished), for PCA analysis normalized spectra were used, thus allowing a qualitative-only analysis (relative content of carotenoids). The normalized spectra are presented in Fig. 5B. A shift of the spectral maxima towards the UV region was observed (the solutions are in DMSO, and thus the maxima were different from those in the HPLC data, where the solvent was different). This shifting of the maxima clearly indicates an increase of β-carotene relative content and decrease of torularhodin relative content as the naftifine concentration was increased. This was in very good agreement with the previously presented chromatographic results (Fig. 4A). Of note, the same approach applied to the bifonazole data did lead to different results. In this case, the decrease in the intensity of carotenoid spectra and increase in antifungal agent concentration were correlated solely with OD decrease (population size of the cells), and no change of the relative concentrations of the various carotenoids was observed. Therefore, the normalized spectra were identical over the entire bifonazole concentration range (Fig. S4), and no clustering was observed when PCA analysis was applied to these spectra (Fig. 5C).

**Figure 6:**
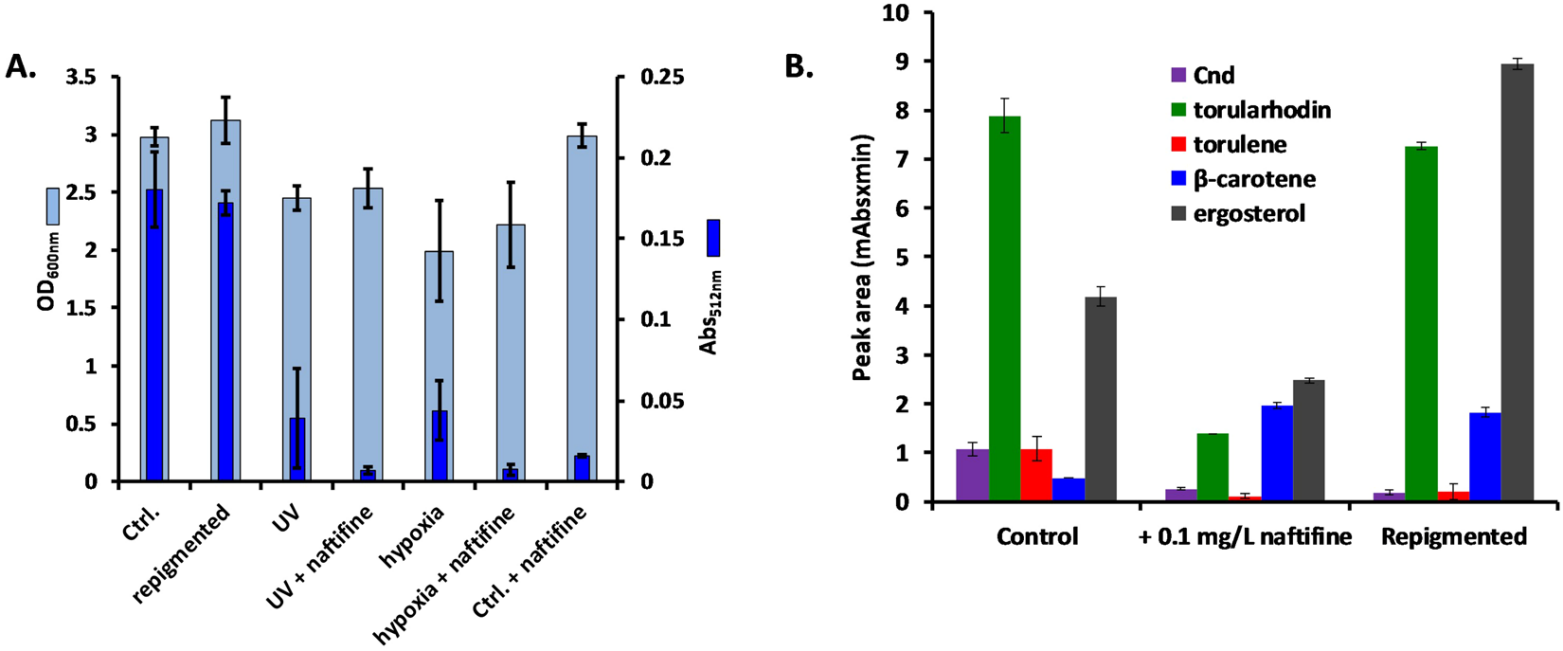
(**A)** OD600nm of the *R. mucilaginosa* cultures cultivated in the indicated conditions and the corresponding Abs at 512 nm after DMSO cell disruption (ANOVA test, p < 0.001 for both sets of histograms). The error bars indicate the standard deviations (n = 3). (**B)** Peak areas of the major components separated and identified in the HPLC chromatogram from Fig. 3A. Ergosterol corresponding values are divided by a factor of 4 to reach the same scale (ANOVA test, p < 0.001).

To further investigate the influence of naftifine upon carotenoid synthesis, we performed experiments where the total carotenoids were monitored by absorbance at 512 nm after DMSO extraction from the cells and by OD_600nm_ to evaluate the cell density (as a marker of cell survival). Contrary to our expectation, hypoxia and UV exposure alone led to depigmentation and had only a weak influence upon cell survival, while the addition of naftifine further suppressed the carotenoid content. Repigmentation of cells treated solely with naftifine (Ctrl. + naftifine) occurred after reinoculation in naftifine-free media (Fig. 6A). A significant profile change in the UV-Vis spectra of the control, repigmented and hypoxia-treated cells was observed by PCA analysis (Fig. 5D). Strikingly, further chromatographic analysis revealed that the relative ratio of the determined carotenoids and ergosterol was very different among the control, naftifine-treated and repigmented forms. The torulene and C_nd_ carotenoids were much lower in the repigmented forms than for the control batch, indicating an alteration of carotenogenesis after naftifine treatment, but β-carotene and ergosterol were much higher (Fig. 6B). This could be of particular importance for biotechnology involving carotenoid synthesis.

**Figure 7:**
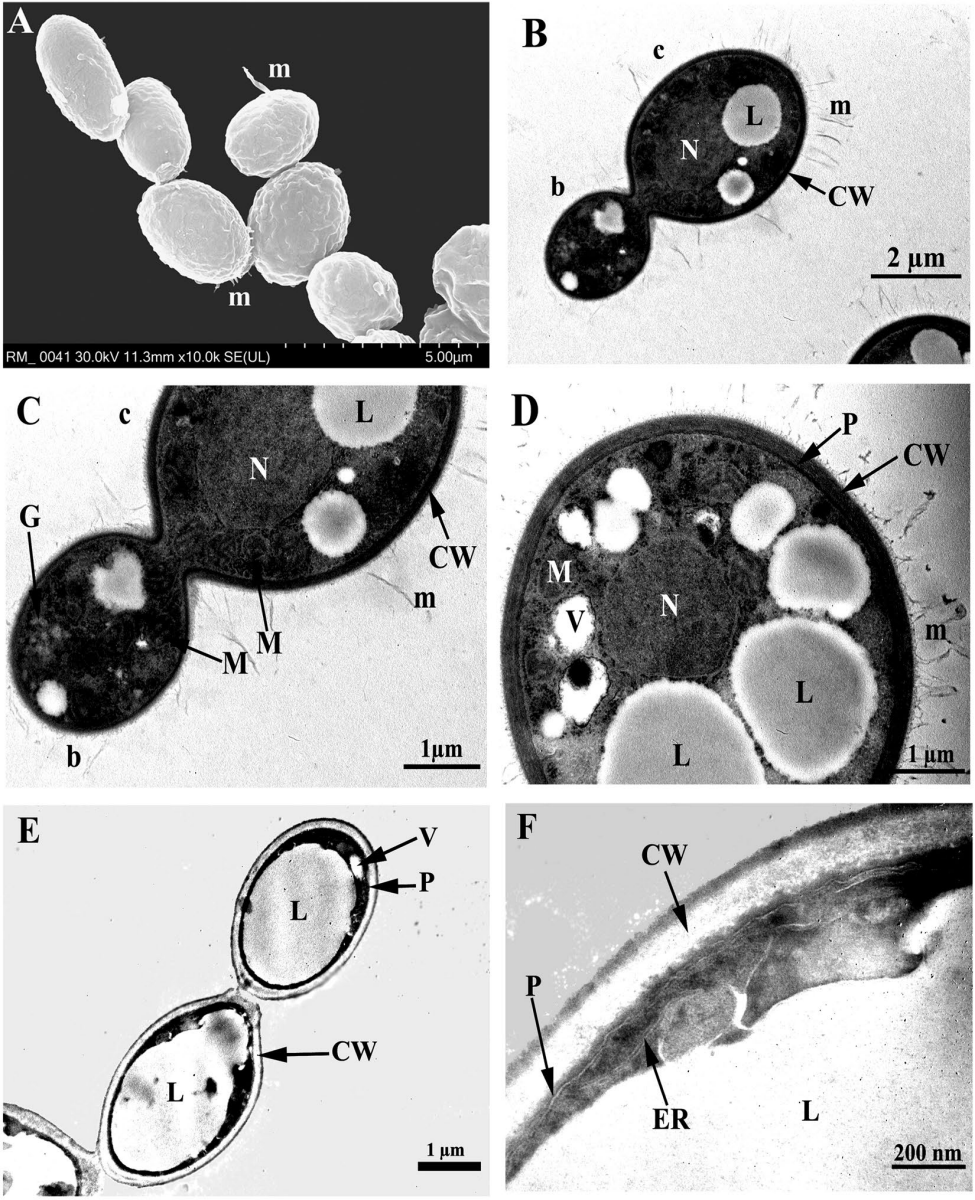
Scanning electron micrograph (**A**) and transmission electron micrographs (**B,C,D,E,F**) of *R. mucilaginosa* showing ultrastructural components. (**A**) Visible mucilage on cell surface; (**B**) longitudinal section of bud and cell; (**C)** longitudinal section of bud and cell (zoomed in); (**D**) cross section of cell; (**E**) longitudinal section of two depigmented cells; (**F)** longitudinal section of a depigmented cell (detailed). Legend: b, bud; c, cell; CW, cell wall; ER, endoplasmic reticulum; G, glycogen; L, lipid; M, mitochondrion; m, mucilage; N, nucleus; P, plasmalemma; V, vacuole.

Following the chemical characterization, morphological and ultrastructural characteristics were evaluated using scanning and transmission electron microscopy. On the cell surface, a characteristic mucilage visible in scanning electron microscopy was observed (Fig. 7A). Ultrastructural characteristics of *R. mucilaginosa* cells include the electron-dense and lamellate cell wall, spherical to ovoid nucleus, plasmalemma, mitochondrion, endoplasmic reticulum, and lipid and glycogen accumulation in the cytoplasm. Numerous small lipid granules of uniform size were found in the cytoplasm of young cells, and larger lipid granules generated by fusion showed variable shapes in mature and senescent cells (Fig. 7B–D). Young *R. mucilaginosa* cells have a thin and electron-dense cell wall formed by elongation of the internal cell wall of the mother cell (Fig. 7B and C). The depigmented cells of *R. mucilaginosa* had a low-electron-density cell wall without visible mucilage or lamellate structure and with large lipid granules (Fig. 7 E and F). *R. mucilaginosa* control colonies were mucoid and pigmented because the cells possess a carotenoid biosynthetic pathway^20, 25^. Additionally, TEM revealed other ultrastructural characteristics as follows. The cells have lamellate and electron-dense cell walls and undergo enteroblastic budding similarly to basidiomycetous yeasts^36, 37^. We suspected that the number of layers of cell wall was correlated with and proportional to the number of buds generated by a cell. In addition to these features, the cells of *R. mucilaginosa* accumulate different storage materials in the main phases of growth, such as glycogen and lipids, similarly to *R. glutinis* ^38^.

Our results complete the list regarding the antifungal effects of naftifine, which provides good activity against *Candida* and *Aspergillus* species^39^ and is an excellent candidate for the treatment of superficial dermatophytosis caused by *Trichophyton rubrum*, *T*. *mentagrophytes*, *T*. *tonsurans*, *Epidermophyton floccosum* and *Microsporum canis* ^40^. For *R. mucilaginosa*, the antifungal activity of naftifine occurs at higher concentrations (MIC = 0.05 mg/L for *R. mucilaginosa*) by preventing ergosterol synthesis^41^.

For the first time, we demonstrated that naftifine caused depigmentation of *R. mucilaginosa* colonies obtained on nutritive medium because the carotenoid biosynthetic pathway was affected. Our results are consistent with the results of other authors, who showed that naftifine has the ability to block the biosynthesis of carotenoids in *Staphylococcus aureus* by affecting the activity of the last enzyme in isoprenoid formation. Moreover, naftifine affected the budding of *R. mucilaginosa* cells, the synthesis of mucilage and the colour and lamellar structure of the cell wall. Bifonazole affected the biosynthetic pathway of ergosterol by inhibiting the demethylation of 4,4′,14-trimethylsterols or by inhibiting the microsomal HMG-CoA-reductase^42^. The carotenoid production was not affected in any way. Amphotericin B binds to ergosterol, determining the formation of pores in the cell membrane, and promotes the accumulation of ROS^43^. The MIC value determined against *R. mucilaginosa* was 1 µg/mL^44^.

## Conclusions

Our research demonstrated that both naftifine (in Exoderil) and bifonazole (in Canespor) were effective antifungal agents for a *R. mucilaginosa* isolate causing onychomycosis, even though they acted differently. Of these fungicides, only naftifine affected the carotenoid synthesis pathway and produced depigmentation in *R. mucilaginosa* cells in a reversible fashion.

Furthermore, naftifine also reduced the relative carotenoid content after repigmentation. Future clinical studies are necessary to further investigate the implications of these findings.

## Methods

### Sample collection

Toenails affected by onychomycosis (Fig. 1A) were obtained from an 85-year-old female with chronic HBV hepatitis. After nail asepsis with 70% ethanol, the distal and lateral fragments of both toenail plates with subungual debris were removed with a sterile nail clipper.

The study was approved by the Ethics Committee of the Iuliu Hatieganu University of Medicine and Pharmacy, Cluj-Napoca (Romania) and an informed written consent was obtained from the patient before enrolling her in the study.

All experiments in this study were performed in accordance with relevant guidelines and regulations.

### Fungal strain isolation and growth conditions

Small toenail fragments of 2–3 mm were disinfected in 20% ethanol for 1 minute and inoculated into SDA control media in Petri dishes^45^. Yeast growth was carried out using the standard method of triple culture (insemination of the inoculum in three points on the surface of SDA in Petri dishes) and incubated at 22 °C for 3 days. After incubation, the isolates were identified by morphological and cultural characteristics. All experiments were performed in triplicate.

Fungal colonies obtained on SDA control media were also grown in Yeast Extract-Peptone-Dextrose (YPD) medium containing 0.5% peptone, 0.3% yeast extract, 0.5% glucose and 30 mg/L chloramphenicol. The media were sterilized at 121 °C for 30 minutes before chloramphenicol addition. Cultures of 10 mL were grown for three days at 22 °C in 50-mL Erlenmeyer flasks on a rotary shaker at 100 rpm (GFL Orbital Shaker 3017, Gesellschaft für Labortechnik mbH, Burgwedel, Germany). In all experiments, the relative yeast growth was evaluated by optical densities at 600 nm (OD_600nm_). Inoculation was performed by adding 50 *µ*L of *R. mucilaginosa* inoculum at 2.4 OD_600nm_ (containing approximate 0.3 × 10^7^ cells) in 10 mL culture media. Before running the experimental tests described in this study, preliminary studies involving OD_600nm_ and Raman profile monitoring were performed to create a growth chart for our experimental conditions, as well as measuring pH changes during the growth of the yeast until maturation.

### Fungal molecular confirmation

The DNA of the *R. mucilaginosa* isolate obtained on SDA control media was extracted using the Animal and Fungi DNA Preparation Kit^®^ (Jena Bioscience) according to the manufacturers’ instructions. The ITS1 and ITS2 primer sets targeting the ITS (Internal Transcribed Spacer) region were used for identification^46^. For PCR amplification, the following mixture was used in a 25 *µ*L final volume: 5 *µ*L of 5X MangoTaq Colored Reaction Buffer (Bioline), 1.25 *µ*L of 50 mM MgCl_2_ (Bioline), 0.5 *µ*L of 10 mM dNTP (Bioline), 1.25 *µ*L of each primer (Macrogen Sequencing Service, Korea), 0.25 *µ*L of 5 U/*µ*L MangoTaq (Bioline) and 2 *µ*L of DNA. Negative control samples were used for each pair of primers. The amplification series consisted of 35 cycles of the following: 95 °C for 30 s, 56 °C for 30 s and 72 °C for 30 s. The fragment obtained after amplification was sequenced at Macrogen (Macrogen Sequencing Service, Korea).

### Antifungal product effects

Two pharmaceutical antifungal products, Exoderil (containing 10 mg/mL of naftifine as active compound, Sandoz GmbH Kundl Austria) and Canespor (containing 10 mg/mL of bifonazole as active compound, KVP Pharma + Veterinär Produkte GmbH, Kiel, Germany), were tested against *R. mucilaginosa*. Minimum inhibitory concentrations (MIC50), the minimum concentrations of compound required to inhibit 50% of the population in terms of OD_600nm_ values, for both antifungal agents was determined in liquid media and expressed in terms of the active compound concentration, followed by comparison with available data^41^. The tested concentrations were 0, 0.005, 0.01, 0.025, 0.05, 0.1, 0.2, 0.6, 1.2, 3.6, 10.8, 50, 100, 500, and 1000 mg/L for naftifine and 0, 0.015, 0.03, 0.06, 0.12, 1, 10, 50, 100, 500, and 1000 mg/L for bifonazole, respectively. Experiments were performed in duplicate.

The *R. mucilaginosa* strain isolated from the SDA control media was used as the inoculum for naftifine effects evaluation. The fungal depigmented colonies were obtained by using inoculum from control *R. mucilaginosa* isolate, exposed for 10 minutes to 1 mg/mL naftifine solution diluted with 20% ethanol, before application to nutritive medium, as inspired by practice recommendations of naftifine hydrochloride dosage and administration^47^. In another experimental variant, the *R. mucilaginosa* depigmented colonies, illustrated by photos, were obtained by including naftifine in small concentrations (0.1 mg/mL, 0.2 mg/mL) in SDA, before inoculation (Fig. 1C). The *R. mucilaginosa* repigmented colonies were obtained from fungal depigmented colonies by inoculation on SDA (Fig. 1D). All fungal colonies obtained on SDA in Petri dishes by method of triple cultures were incubated at 22 °C for 3 days, and all the experiments were performed in triplicate.

The concentration of 1.2 mg/L naftifine in YPD medium was chosen for subsequent investigations. An inoculum of 20 *µ*L was used for 10 mL culture media. Two sets of experiments were carried out for *R. mucilaginosa* inoculated in YPD medium without naftifine and with naftifine. *R. mucilaginosa* previously treated with 1.2 mg/L naftifine was also used for inoculation in YPD medium without naftifine. For the first experiment, after inoculation the Erlenmeyer flasks were exposed to UV radiation for 40 minutes in a biological safety cabinet. For the second (hypoxic) experiment, flasks inoculated with *R. mucilaginosa* were sealed after argon was bubbled for 60 s in each flask. These tests were performed in quintuplicate. The results were reproduced in independent trials.

### Carotenoid evaluation using UV-vis and resonant Raman spectroscopies

Cells from 2 mL of homogenous suspension were harvested and washed three times with 1 mL 0.9% NaCl solution; 100 *µ*L of the final suspension in saline solution were used for Raman spectroscopy, while the other 900 *µ*L were centrifuged and the saline solution was discarded. The pellet was resuspended in 1.8 mL of DMSO, immediately followed by vigorous shaking for complete cell disruption (transformation of the suspension into a clear solution) and carotenoid extraction. The clear solution was further centrifuged at 20000 g speed for cell debris deposition. The UV-vis spectra of the clear supernatant were measured between 200 and 800 nm using a Varian Cary 5000 UV-Vis-NIR Spectrophotometer (Agilent Technologies). Absorbance at 512 nm (maximum wavelength for these samples) was taken as a marker of the total carotenoid content.

Resonant Raman spectra were recorded using a confocal Renishaw inVia Reflex Raman Spectrometer at 5% of the 200 mW Cobolt Diode Pumped Solid State (DPSS) laser, emitting at 532 nm. The Raman back-scattered light was directed to a spectrometer equipped with 1800 lines/mm grating and a CCD detector. The spectral resolution was ~4 cm^−1^. The instrument was calibrated prior to each experiment using a silicon sample as an internal standard. Cells in saline buffer were applied to poly-L-lysine adhesive slides (Polysine™ Microscope Adhesion Slides, Erie Scientific via VWR) for efficient sample immobilization. The laser was focused on the sample using a 100x objective, and the spectra were recorded with 10-s exposure times and 2 accumulations. The experiments were done in triplicate.

Resonant Raman maps were obtained by raster-scanning the selected area with the 532-nm laser, focused on the sample using a 5x objective. The laser power was set to 10%. The exposure time for each spectrum was 2 s and the measurement step was 200 *µ*m. The Raman signal contours map, as a function of the Raman intensity, is shown in Fig. 4C.

### Chromatographic analysis

Both HPLC and HPTLC approaches were used for separating and evaluating the relative quantity of the carotenoids and ergosterol in *R. mucilaginosa* after DMSO cell disruption. An Agilent 1200 HPLC system (Waldbronn, Germany) equipped with an on-line vacuum degasser, quaternary pump, temperature-controlled sample tray, automatic injector, column thermostat compartment and DAD detector was employed, with a Nucleosil 100 C18 column (240 mm × 4.6 mm, 5 *µ*m particle size) from Macherey-Nagel (Duren, Germany). The injection volume was 100 *µ*L (0.2 *µ*m filtered DMSO extract), the column temperature was set to 25 °C and the flow rate was 1.2 mL/minute. As a standard, β-carotene at a concentration of 1 mg/mL was used. An isocratic elution was employed using acetonitrile:methanol:isopropanol at 85:10:5, as previously described^27^. UV-Vis detection of compounds was accomplished using the DAD detector to measure the entire spectrum in the 190–700 nm range every 1 s, and the chromatograms were monitored at 254, 450, 490 and 512 nm. The chromatograms were exported and the graphs were developed in Excel and Origin 6, where the integration of the peaks was also performed. HPTLC was performed on HP silica gel plates (Silica gel 60, Merck, Darmstadt, Germany) using hexane-acetone-methanol (10:15:75 v/v/v) as the mobile phase.

### Electron microscopy

*R. mucilaginosa* cells were examined using scanning electron microscopy (SEM) with a JEOL JSM 5510 LV electron microscope and transmission electron microscopy (TEM) with a JEOL JEM 1010 electron microscope (Japan Electron Optics Laboratory Co., Tokyo, Japan). The samples were fixed in 2.7% glutaraldehyde (in phosphate-buffered saline for 90 minutes). For SEM, the samples were critical-point dried in liquid CO_2_, mounted on sticky carbon tabs and sputter-coated with gold (10 nm). For TEM, the fixed and dried samples were infiltrated with resin (Epon 812), deposited onto colloidal-carbon-coated copper grids and negatively stained with lead citrate and uranyl acetate^48^. The chemicals needed for electron microscopy experiments were purchased as follows: glutaraldehyde, resin (Epon 812), lead citrate, uranyl acetate, bismuth subnitrate (Electron Microscopy Sciences, Fort Washington, USA); sticky carbon tabs, colloidal carbon coated grids (Agar Scientific, Cambridge, England). Details regarding the sample work protocol for electron microscopy were presented in other reports^49^.

### Data analysis

Statistical analysis was performed using Statistica 7.0 for Windows (Stat-Soft, Inc., USA) and Excel. Principal component analysis (PCA) was applied to several data sets as indicated in the text using Statistica 7.0. T-tests were used to test the strength of association within the data. Where appropriate, using p < 0.05 as threshold for statistical significance, a statistical approach was formulated and the experimental data were also evaluated using one-way analysis of variance (ANOVA). The statistical parameters confirm the hypothesis that the differences between the results are either not significant (p > 0.05), significant (0.001 < p < 0.05) or highly significant (p < 0.001). The average of multiple measurements (duplicates, triplicates or more, depending on type of measurement) was used for plots, and the error bars are the standard error of the mean. For MIC determination, Origin 6 was used to fit the data and determine the MIC50.

### Data Availability

The datasets generated during and/or analysed during the current study are available from the corresponding author on reasonable request.

## Electronic supplementary material

Supplementary data
