# Supplementary material for: Reversible naftifine-induced carotenoid depigmentation in Rhodotorula mucilaginosa (A. Jörg.) F.C. Harrison causing onychomycosis
Source: Sci Rep. 2017 Sep 11;7:11125. doi: 10.1038/s41598-017-11600-7 (PMC5593942; doi:10.1038/s41598-017-11600-7)
Supplement: Supplementary file 1 — Supplementary data [file 41598_2017_11600_MOESM1_ESM.pdf]

Supplementary data

Reversible naftifine-induced carotenoid depigmentation in *Rhodotorula mucilaginosa* (A. Jörg.) F.C. Harrison causing onychomycosis

Augustin C. Moț[1], Marcel Pârvu[2*], Alina E. Pârvu[3], Oana Roşca-Casian[4], Nicoleta E. Dina[5], Nicolae Leopold[6], Radu Silaghi-Dumitrescu[1], Cristina Mircea[2]

[1] Babeș-Bolyai University, Faculty of Chemistry and Chemical Engineering, 11 Arany Janos Street, 400028 Cluj-Napoca, Romania
[2] Babeș-Bolyai University, Faculty of Biology and Geology, 42 Republicii Street, 400015 Cluj-Napoca, Romania
[3] Iuliu Hatieganu University of Medicine and Pharmacy, Faculty of Medicine, Department of Pathophysiology, 3 Victor Babes Street, 400012 Cluj-Napoca, Romania
[4] Babeș-Bolyai University, Alexandru-Borza Botanical Garden, 42 Republicii Street, 400015 Cluj-Napoca, Romania
[5] National Institute for Research and Development of Isotopic and Molecular Technologies, 67-103 Donath Street, 400293 Cluj-Napoca, Romania
[6] Babeș-Bolyai University, Faculty of Physics, 1 Mihail Kogalniceanu Street, 400005 Cluj-Napoca, Romania

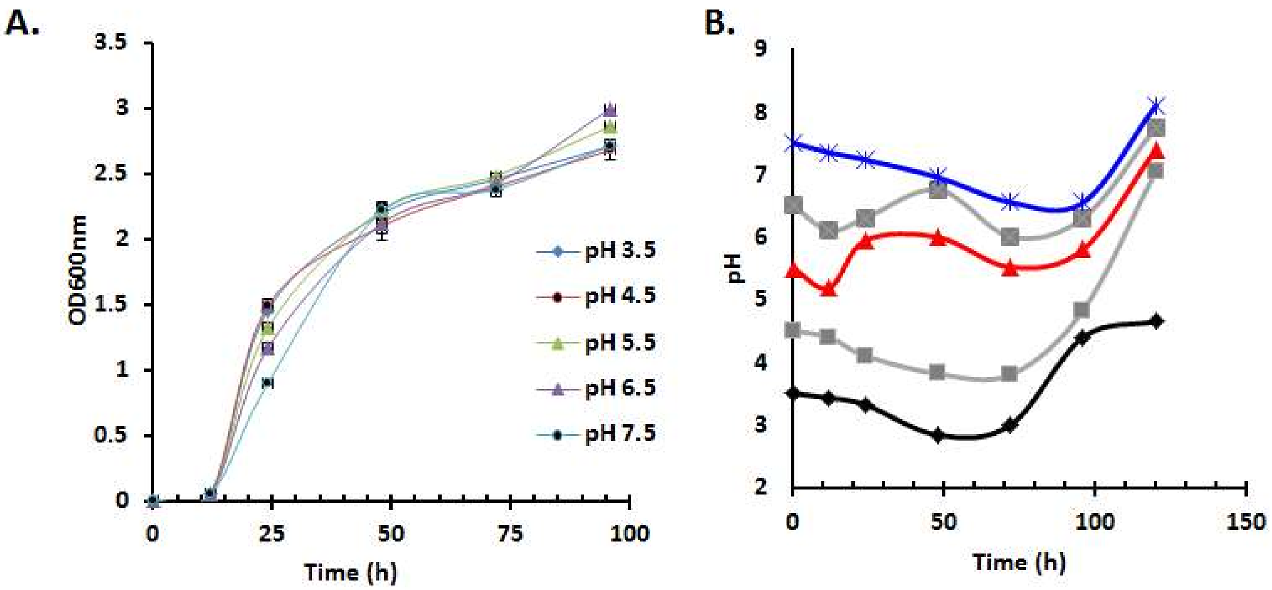

Figure S1. A. Growth of *R. mucilaginosa* at various initial pH values (of the culture medium). **B.** pH change in time for the various initial pH values of the culture medium.

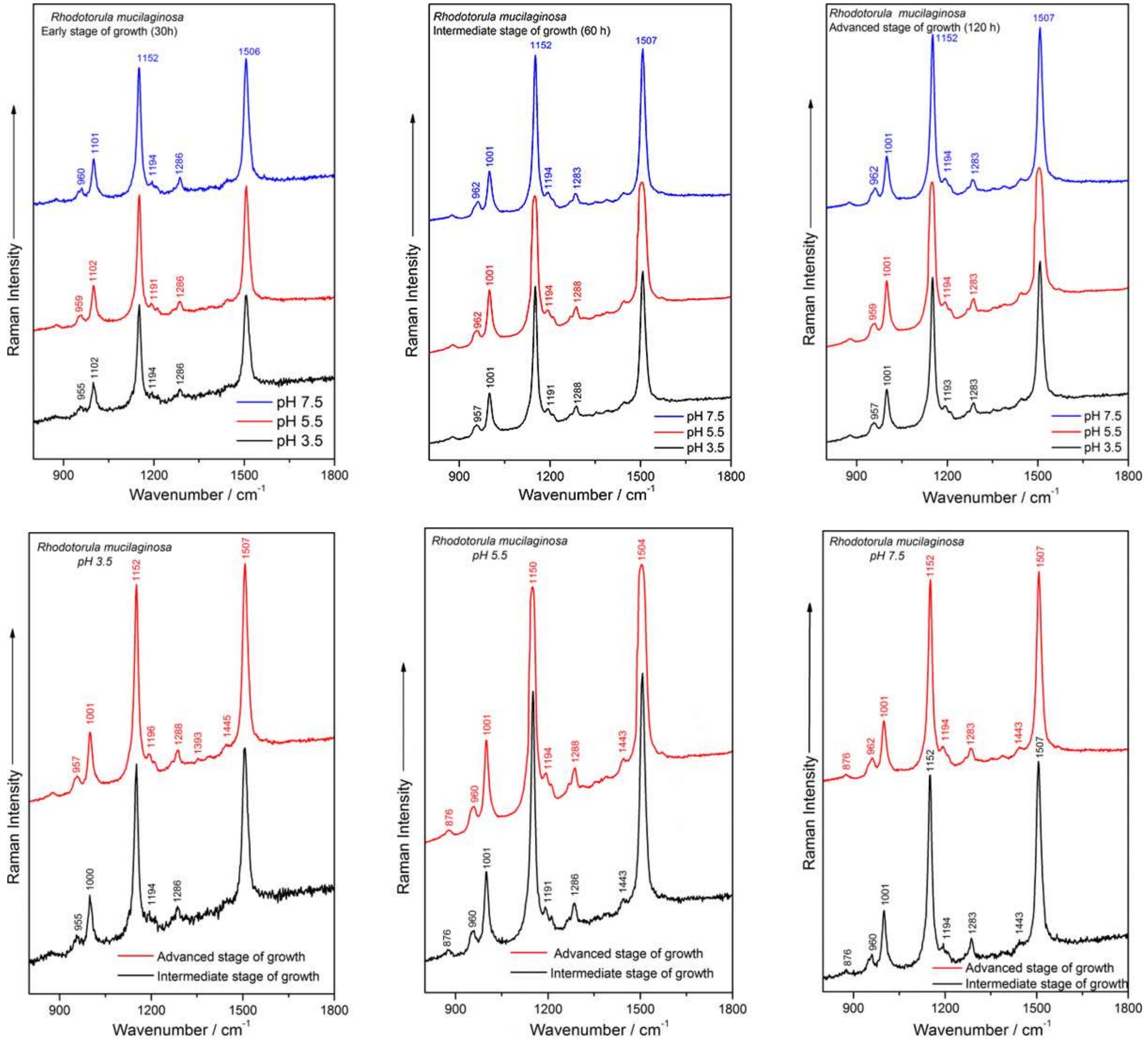

Figure S2. Resonant Raman monitoring of *R. mucilaginosa* cells, at three different growth stages (early (30h), intermediate (60h) and advanced (120h)), at three different pH values.

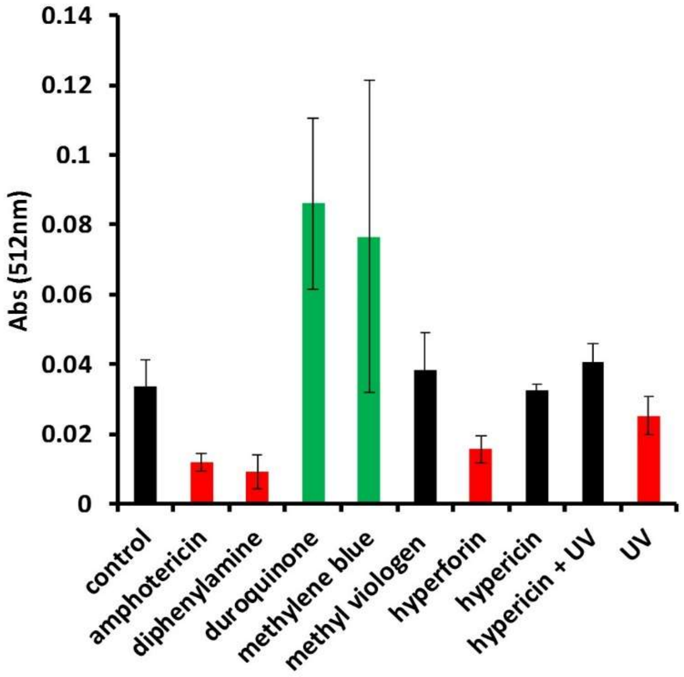

Figure S3. A series of chemicals were tested against *Rhodotorula mucilaginosa* in order to evaluate their effect on carotenoid production. Duroquinone, methylene blue and methyl viologen induce oxygen reactive species; hypericin and hyperforin are photosensitizers, while diphenylamine and amphotericin B inhibit carotenoid formation and fungal growth, respectively. The chemicals were tested at final concentrations of: 16.4 μg/mL for duroquinone, 0.034 μg/mL for methylene blue, 0.002 μg/mL for methyl viologen, 12.6 μg/mL for hypericin, 5.36 μg/mL for hyperforin, 0.84 μg/mL for diphenylamine and 1 μg/mL for amphotericin B, according to literature recommendations for their maximum activity (where available). For the concentration of 12.6 μg/mL of hypericin, an additional set of experiments were carried out. The Falcon tubes were exposed, after the inoculation, to UV radiation for 15 minutes in a biological safety cabinet. The same irradiation conditions were applied to *R. mucilaginosa* cells not treated with any chemical. The tests were performed in triplicate.

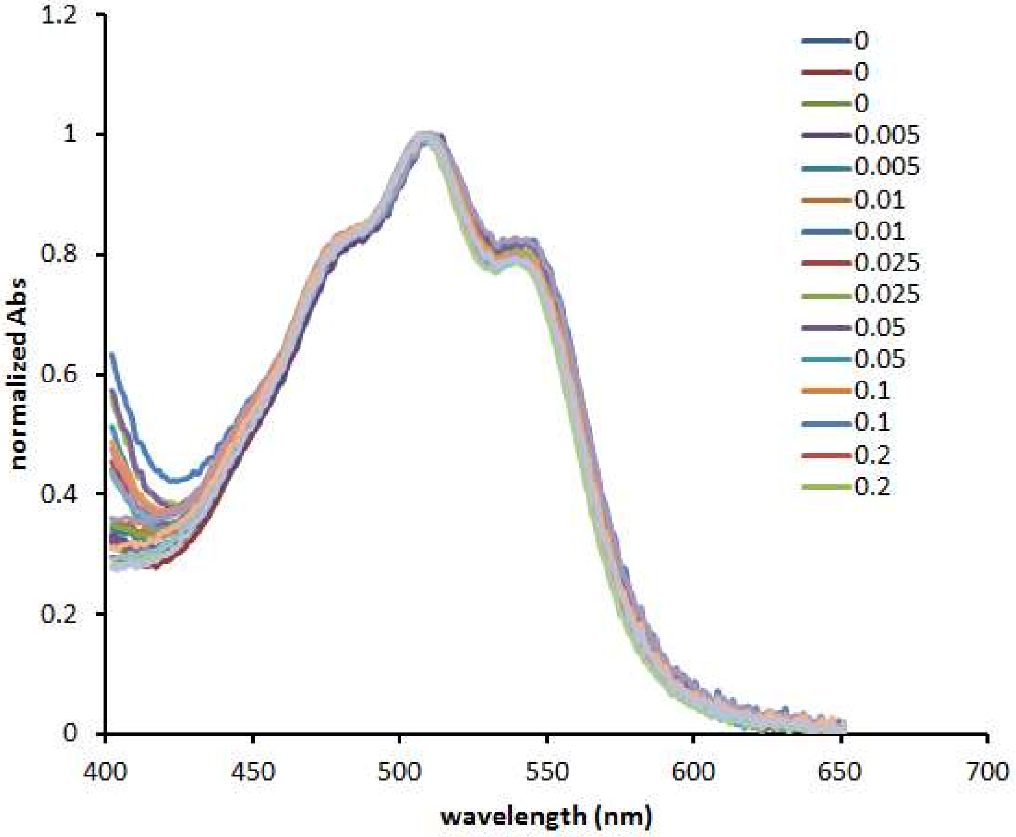

Figure S4. The molecular absorbance UV-vis spectra used in the PCA analysis at specific bifonazole working concentration (mg/L).
